# Validation of the 21-gene test as a predictor of clinical response to neoadjuvant hormonal therapy for ER+, HER2-negative breast cancer: the TransNEOS study

**DOI:** 10.1007/s10549-018-4964-y

**Published:** 2018-09-21

**Authors:** Hiroji Iwata, Norikazu Masuda, Yutaka Yamamoto, Tomomi Fujisawa, Tatsuya Toyama, Masahiro Kashiwaba, Shoichiro Ohtani, Naruto Taira, Takehiko Sakai, Yoshie Hasegawa, Rikiya Nakamura, Hiromitsu Akabane, Yukiko Shibahara, Hironobu Sasano, Takuhiro Yamaguchi, Kentaro Sakamaki, Helen Bailey, Diana B. Cherbavaz, Debbie M. Jakubowski, Naoko Sugiyama, Calvin Chao, Yasuo Ohashi

**Affiliations:** 1 Aichi Cancer Center Hospital, 1-1 Kanokoden, Chikusa-ku, Nagoya, Aichi 464-0021 Japan; 2 NHO Osaka National Hospital, 2-1-14 Hoenzaka, Chuo-ku, Osaka, 540-0006 Japan; 3 Kumamoto University, 1-1-1 Honjo, Chuo-ku, Kumamoto, 860-8556 Japan; 4 Gunma Prefectural Cancer Center, 617-1 Takahayashinishi-cho, Ota, Gunma 373-8550 Japan; 5 Nagoya City University Graduate School of Medical Science, 1 Kawasumi, Mizuho-cho, Mizuho-ku, Nagoya, Aichi 467-8601 Japan; 6 Sagara Hospital, 3-31 Matsubara-cho, Kagoshima, 892-0833 Japan; 7 Hiroshima City Hiroshima Citizens Hospital, 7-33 Motomachi, Naka-ku, Hiroshima 730-8518 Japan; 8 Okayama University Hospital, 2-5-1 Shikata-cho, Kita-ku, Okayama, 700-8558 Japan; 9 Cancer Institute Hospital of Japanese Foundation for Cancer Research, 3-8-31 Ariake, Koto-ku, Tokyo, 135-8550 Japan; 10 Hirosaki Municipal Hospital, 3-8-1 Oaza Omachi, Hirosaki, Aomori 036-8004 Japan; 11 Chiba Cancer Center, 666-2, Nitona-cho, Chuo-ku, Chiba Japan; 12 Hokkaido P.W.F.A.C. Asahikawa-Kosei General Hospital, 24-111 Ichijo-dori, Asahikawa, Hokkaido 078-8211 Japan; 13 Tohoku University Graduate School of Medicine, 1-1 Seiryo-machi, Aoba-ku, Sendai, Miyagi 980-8574 Japan; 14 Graduate School of Medicine, The University of Tokyo, 7-3-1, Hongo, Bunkyo-ku, Tokyo, 113-8655 Japan; 15 Genomic Health, Inc., 301 Penobscot Dr, Redwood City, CA USA; 16 Chuo University, 1-13-27 Kasuga, Bunkyo-ku, Tokyo, 112-8551 Japan

**Keywords:** Breast cancer, Hormonal therapy, Neoadjuvant, Oncotype DX, Recurrence score

## Abstract

**Purpose:**

The Recurrence Score test is validated to predict benefit of adjuvant chemotherapy. TransNEOS, a translational study of New Primary Endocrine-therapy Origination Study (NEOS), evaluated whether Recurrence Score results can predict clinical response to neoadjuvant letrozole.

**Methods:**

NEOS is a phase 3 clinical trial of hormonal therapy ± adjuvant chemotherapy for postmenopausal patients with ER+, HER2-negative, clinically node-negative breast cancer, after six months of neoadjuvant letrozole and breast surgery. TransNEOS patients had tumors ≥ 2 cm and archived core-biopsy samples taken before neoadjuvant letrozole and subsequently sent for Recurrence Score testing. The primary endpoint was to evaluate clinical (complete or partial) response to neoadjuvant letrozole for RS < 18 versus RS ≥ 31. Secondary endpoints included evaluation of clinical response and rate of breast-conserving surgery (BCS) by continuous Recurrence Score result, *ESR1* and *PGR* single-gene scores, and ER gene-group score.

**Results:**

Of 295 TransNEOS patients (median age 63 years; median tumor size 25 mm; 66% grade 1), 53.2% had RS < 18, 28.5% had RS18–30, and 18.3% had RS ≥ 31. Clinical response rates were 54% (RS < 18), 42% (RS18–30), and 22% (RS ≥ 31). A higher proportion of patients with RS < 18 had clinical responses (*p* < 0.001 vs. RS ≥ 31). In multivariable analyses, continuous Recurrence Score result (*p* < 0.001), *ESR1* score (*p* = 0.049), *PGR* score (*p* < 0.001), and ER gene-group score (*p* < 0.001) were associated with clinical response. Recurrence Score group was significantly associated with rate of BCS after neoadjuvant treatment (RS < 18 vs. RS ≥ 31, *p* = 0.010).

**Conclusion:**

The Recurrence Score test is validated to predict clinical response to neoadjuvant letrozole in postmenopausal patients with ER+, HER2-negative, clinically node-negative breast cancer.

**Electronic supplementary material:**

The online version of this article (10.1007/s10549-018-4964-y) contains supplementary material, which is available to authorized users.

## Introduction

Neoadjuvant therapy for locally advanced breast cancer has the potential to improve rates of breast-conserving surgery (BCS), permit the assessment of patients’ primary tumor response to systemic therapy, and reduce rates of distant metastases by as much as that observed with adjuvant approaches [1–6]. Clinical and pathological complete responses (CR) to neoadjuvant therapy are associated with improved clinical outcomes, and both are considered to be valid surrogates of clinical outcomes for some types of breast cancer [1, 6, 7].

Previous studies have demonstrated higher rates of clinical and pathological CR to neoadjuvant chemotherapy in high-grade tumors and tumors with higher levels of Ki-67 protein expression, but lower rates of response in tumors positive for estrogen receptor (ER) protein expression [7–9]. Historically, neoadjuvant hormonal therapy was reserved only for patients who were not candidates for neoadjuvant chemotherapy or surgery. Studies conducted since 2001, however, have demonstrated that neoadjuvant hormonal therapy can yield clinically meaningful response rates in more general populations of patients with ER + breast cancer [10–16]. Therefore, many patients with locally advanced ER + breast cancer may be considered for neoadjuvant hormonal therapy, as a viable means to achieve clinical response and improve rates of BCS [12–19]. As in the adjuvant setting, however, response to neoadjuvant hormonal therapy can vary across patients with ER + breast cancer [20]. Thus, the capacity to select patients who are more likely to benefit from neoadjuvant hormonal therapy would represent an advance in the clinical management of breast cancer.

The Oncotype DX Breast Recurrence Score® test is a validated clinical tool that predicts benefit of adjuvant chemotherapy in patients with ER+, HER2-negative, node-negative [21–25], and node-positive [24, 26] early breast cancer who receive five years of hormonal therapy. In addition, the Recurrence Score test is validated to predict distant recurrence in patients treated with adjuvant hormonal therapy. The clinical validation study used samples from the National Surgical Adjuvant Breast and Bowel Project (NSABP) B-14 trial to show that the Recurrence Score® (RS) results quantified the likelihood of distant recurrence in patients with node-negative, ER + breast cancer who received tamoxifen [21].

Previous studies suggested that the Recurrence Score test might be useful in the neoadjuvant setting. Studies have demonstrated a relationship between the Recurrence Score result and response to neoadjuvant chemotherapy, with clinical or pathological CR more likely achieved in patients with higher Recurrence Score results [27–33]. Other studies have shown a correlation between low Recurrence Score results (RS < 18) and greater likelihood of response to neoadjuvant hormonal therapy [34, 35]. In one study of 43 postmenopausal women with ER+, progesterone receptor-positive (PgR+) breast cancer who received neoadjuvant tamoxifen or anastrozole for four months, the rate of clinical response [CR + PR (partial response)] was 64%, 31%, and 31% for patients with RS < 18, RS18–30, and RS ≥ 31, respectively [34]. In another study of 64 women with ER + breast cancer who received 16–24 weeks of neoadjuvant exemestane, the rate of clinical response was 59% for patients with RS < 18 and 20% for patients with RS ≥ 31 (*p* = 0.015). Rates of BCS were 91% with RS < 18 and 47% with RS ≥ 31 (*p* = 0.003) [35]. Together, these study findings suggest that the Recurrence Score test could have utility in selecting patients for appropriate neoadjuvant therapy.

The New Primary Endocrine-Therapy Origination Study (NEOS), a phase 3 trial initiated in 2008, evaluated disease-free survival of postmenopausal patients with ER+, HER2-negative, node-negative, non-metastatic primary breast cancer who were randomized to adjuvant hormonal therapy with or without chemotherapy based on clinical response (CR, PR, or stable disease [SD]) to 24–28 weeks of neoadjuvant letrozole treatment. Surgery was performed before initiation of specified adjuvant treatment (Supplemental Fig. 1) [36]. Herein we report the findings of TransNEOS, a translational study of NEOS that was prospectively designed to evaluate the utility of the Recurrence Score test to predict clinical response to neoadjuvant hormonal therapy and successful BCS in postmenopausal women with ER+, HER2-negative, clinically node-negative breast cancer.

## Methods

### Study patients

Patients eligible for the TransNEOS study were previously enrolled in the parent NEOS study and were recruited from the 25 NEOS study centers with the highest patient enrollment. Eligible patients gave informed consent. They were postmenopausal women < 75 years of age with T1c-T2 (≥ 2 cm in the largest dimension, as measured by magnetic resonance imaging [MRI] and/or computed tomography scan), clinically node-negative, non-metastatic, ER+, HER2-negative invasive breast cancer who received neoadjuvant letrozole. Eligible patients had core-biopsy samples taken before neoadjuvant treatment that were later sent for Recurrence Score testing (Genomic Health, Inc., Redwood City, CA). Patients with no Recurrence Score results, no tumor block available, no or insufficient tumor in block as determined by a pathologist at Genomic Health in accordance with standard operating procedures, or insufficient or inadequate RNA for testing were excluded. Unique patient and specimen identifiers were assigned by the principal investigator and blinded to Genomic Health.

### Study protocol

All patients included in the TransNEOS study received 24–28 weeks of neoadjuvant letrozole per the NEOS study protocol. Clinical response to neoadjuvant letrozole was assessed by mono-dimensional measurement of the largest diameter of the target tumor by MRI or computed tomography scan at baseline and at the conclusion of neoadjuvant treatment. Complete response (CR) was defined if the target tumor disappeared, partial response (PR) if the largest diameter of the target tumor was reduced by ≥ 30% from baseline, stable disease (SD) if the largest diameter of the target tumor was reduced by < 30% or increased by < 20% from baseline, and progressive disease (PD) if the largest diameter of the target tumor increased by ≥ 20% from baseline. Clinical response rate was defined as the sum of the rates of CR and PR. Recurrence Score testing was performed in a central laboratory (Genomic Health, Inc., Redwood City, CA) on RNA extracted from formalin-fixed paraffin-embedded core-biopsy samples, as previously described [21]. Recurrence Score results, single-gene results for estrogen receptor (*ESR1*), progesterone receptor (*PGR*), and HER2 (*ERBB2*), and gene-group scores for the ER gene-group (*ESR1, PGR, BCL2, SCUBE2*) and proliferation gene-group (*BIRC5, MKI67, MYBL2, CCNB1, AURKA*) were reported as values of the Recurrence Score test. Standard Recurrence Score risk categories were used: RS < 18, RS18–30, and RS ≥ 31. Both local and central pathology determinations of ER (immunohistochemistry [IHC] or Allred score), PgR (IHC or Allred score), and HER2 (IHC and/or fluorescent in situ hybridization), and central pathology determination of Ki-67 (IHC) were performed on all tumor samples using standard techniques. BCS candidacy before neoadjuvant treatment was determined by the treating surgeon. The study was reviewed and approved by each institution’s review board and/or ethics committee.

### Study endpoints

The primary endpoint was to compare the clinical response (CR + PR) to neoadjuvant letrozole between patients with RS < 18 and RS ≥ 31. Secondary clinical endpoints included description of the relationship between the clinical response to neoadjuvant letrozole and (a) continuous Recurrence Score result; (b) *ESR1, PGR*, and ER gene-group scores by RT-PCR; (c) Ki-67 by IHC; and (d) proliferation gene-group score by RT-PCR. In addition, correlations between Ki67 by IHC and (a) Recurrence Score results and (b) proliferation gene-group score by RT-PCR were examined.

Secondary surgical endpoints included description of the relationship between the rate of BCS after neoadjuvant letrozole and (a) continuous Recurrence Score result; (b) categorical Recurrence Score groups (RS < 18 and RS ≥ 31); and (c) *ESR1, PGR*, and ER gene-group scores by RT-PCR. In addition, the relationships between change in treatment from mastectomy to BCS after neoadjuvant letrozole and (a) continuous Recurrence Score result and (b) categorical Recurrence Score groups (RS < 18 and RS ≥ 31) were evaluated.

### Statistical analysis

All analyses were prespecified and included patients with reportable values of the associated variables. All hypothesis tests were reported using two-sided *p* values, and *p* values < 0.05 were considered statistically significant. The clinical and pathological characteristics of the patients were reported descriptively. Continuous variables were summarized as mean (standard deviation) and/or median (range). Categorical variables were reported as numbers and proportions (percentage). A *χ*^2^ test was used to determine if the proportion of patients with clinical response in the RS < 18 group was significantly higher than the proportion with clinical response in the RS ≥ 31 group. For small expected group counts, Fisher’s exact test was used. A logistic regression model was used to describe the relationship between clinical response and the Recurrence Score result, the continuous *ESR1* result by RT-PCR, the continuous *PGR* result by RT-PCR, the ER gene-group score, the proliferation gene-group score, and Ki-67 percent-staining. Single-gene and gene-group scores were standardized to represent the proportional increase in odds of one standard deviation. These analyses were performed with and without adjustment for clinical covariates. The Spearman correlation between Ki-67 percent-staining and Recurrence Score result (95% confidence interval [CI]), and between Ki-67 percent-staining and proliferation gene-group score (95% CI) were reported. The proportion of patients in the RS < 18, RS18–30, and RS ≥ 31 groups who were candidates for BCS before and who actually received BCS after neoadjuvant treatment, and who changed treatment from mastectomy to BCS before and after neoadjuvant treatment, was calculated. (Information about BCS candidacy after neoadjuvant treatment was not collected, only actual surgery received.) A logistic regression model was used to describe the relationship between Recurrence Score group and (a) changes in BCS candidacy before neoadjuvant treatment; and (b) BCS received after neoadjuvant treatment. A logistic regression model was used to describe the relationship between the rate of BCS after neoadjuvant treatment and (a) continuous *ESR1* result by RT-PCR; (b) continuous *PGR* result by RT-PCR; and (c) continuous ER gene-group score by RT-PCR. These analyses were performed with and without adjustment for clinical covariates. As *ESR1* and *PGR* are components of the Recurrence Score result, each variable was considered in separate models to avoid issues with collinearity. All analyses were performed in SAS 9.4 (SAS Institute Inc., Cary, NC).

## Results

### Characteristics of the patients

**Fig. 1:**
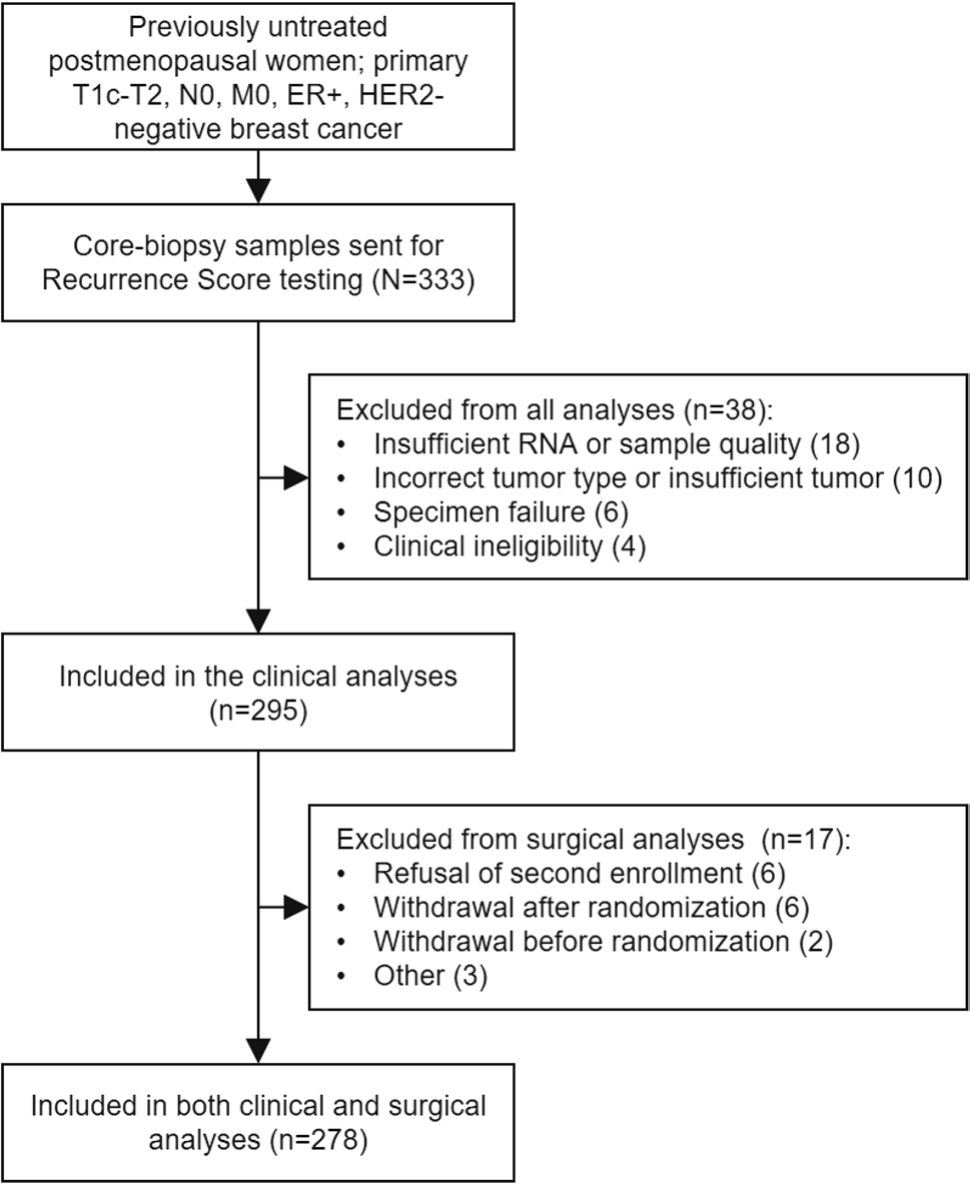
REMARK diagram

In total, 333 core-biopsy samples were submitted for Recurrence Score testing. Of these, 38 samples were excluded for reasons of insufficient RNA or sample quality (*n* = 18), incorrect tumor type or insufficient tumor (*n* = 10), specimen failure (*n* = 6), and clinical ineligibility (*n* = 4) (Fig. 1). Characteristics of the TransNEOS study cohort (*N* = 295) are summarized in Table 1. Median Recurrence Score result was RS17 (range RS0–68); 157 (53.2%) had RS < 18, 84 (28.5%) had RS18–30, and 54 (18.3%) had RS ≥ 31.

**Table 1:** Patient demographics and disease characteristics (*N* = 295)

Variable	Statistic	Result
Age, years	Median (range)	63 (49–75)
	≤ 60	94 (31.9%)
	> 60 to 70	169 (57.3%)
	> 70	32 (10.8%)
T-stage	T1c	44 (14.9%)
	T2	251 (85.1%)
Nuclear grade	1	195 (66.1%)
	2	59 (20.0%)
	3	27 (9.2%)
	Missing	14 (4.7%)
Tumor size, mm	Median (range)	25 (20–65)
Ki-67 by IHC, %	Median (range)	16.2 (0.0, 82.5)
Ki-67 category	< 10%	86 (29.2%)
	10–30%	123 (41.7%)
	> 30%	61 (20.7%)
	Missing	25 (8.5%)
*ESR1* by RT-PCR	Median (range)	11.7 (5.7–14.6)
	Mean (st dev)	11.5 (1.3)
ER category, determined by RT-PCR	Positive (≥ 6.5)	293 (99.3%)
	Negative (< 6.5)	2 (< 1.0%)
*PGR* by RT-PCR	Median (range)	7.1 (2.6–11.4)
	Mean (st dev)	6.7 (2.0)
PgR category, determined by RT-PCR	Positive (≥ 5.5)	211 (71.5%)
	Negative (< 5.5)	84 (28.5%)
HER2 category, determined by RT-PCR	Negative (< 10.7)	235 (79.7%)
	Positive (≥ 11.5)	9 (3.1%)
	Equivocal (10.7 to < 11.5)	51 (17.3%)
Recurrence Score result	Median (range)	17 (0–68)

*ER* estrogen receptor, *HER2* human epidermal growth factor receptor 2, *IHC* immunohistochemistry, *PgR* progesterone receptor, *RT-PCR* reverse transcription-polymerase chain reaction, *st dev* standard deviation

### Clinical response to neoadjuvant letrozole by Recurrence Score group

**Fig. 2:**
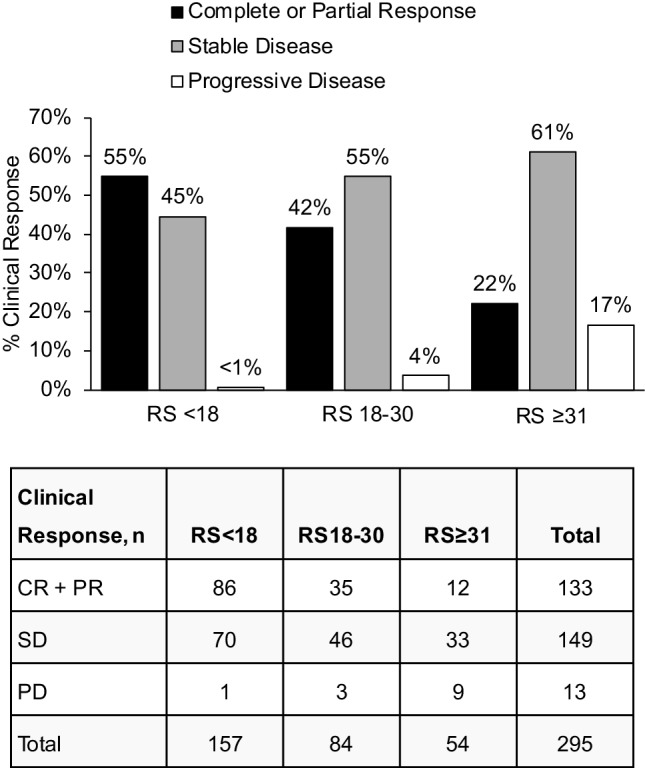
Percent clinical response to neoadjuvant letrozole by Recurrence Score Group (*N* = 295); *CR* complete response, *PD* progressive disease, *PR* partial response, *RS* Recurrence Score, *SD* stable disease

The prespecified primary endpoint was met: Recurrence Score group (RS < 18 vs. RS ≥ 31) was significantly associated with the rate of clinical response (*χ*^2^ test, *p* < 0.001). Among patients with RS < 18, RS18–30, and RS ≥ 31, rate of clinical response was 55%, 42%, and 22%, respectively. With the RS18–30 group included, Recurrence Score group remained significantly associated with the rate of clinical response (Cochran–Armitage trend test, *p* < 0.001). At < 1%, patients with RS < 18 had the lowest rate of PD, compared with patients with RS18–30 (4% PD) and RS ≥ 31 (17% PD) (Fisher’s exact test, *p* < 0.001; Fig. 2).

### Univariable analysis of clinical response to neoadjuvant letrozole

Univariable logistic regression analysis showed that continuous Recurrence Score result (*p* < 0.001), *ESR1* result by RT-PCR (*p* = 0.019), *PGR* result by RT-PCR (*p* < 0.001), and ER gene-group score (*p* < 0.001) were significant predictors of clinical response to neoadjuvant letrozole (Supplemental Table 1). Neither the proliferation gene-group score nor Ki-67 percent-staining by IHC predicted clinical response (Supplemental Table 1).

### Multivariable analysis of clinical response to neoadjuvant letrozole, controlling for clinical factors

In a multivariable logistic regression model, the continuous Recurrence Score result remained a significant predictor of clinical response after controlling for patient age, tumor size, and tumor grade (Table 2); odds ratio (95% CI) was 0.06 (0.02 to 0.18) (profile likelihood test, *p* < 0.001). Because *ESR1* by RT-PCR, *PGR* by RT-PCR, and ER gene-group score represent single-variable (individual) components of the Recurrence Score algorithm, each was considered individually in separate multivariable analyses (Table 3). In separate multivariable models controlling for age, tumor size, and tumor grade, *ESR1* by RT-PCR, *PGR* by RT‑PCR, and ER gene-group score were significant predictors of clinical response. Both *PGR* by RT-PCR and ER gene-group score were stronger predictors of clinical response than *ESR1* by RT-PCR (Table 3).

**Table 2:** Multivariable analysis^a^ of clinical response, controlling for clinical factors (*N* = 281)

Variable	Odd ratio (95% CI)	*p* value*
Recurrence Score result (per 50-unit increase^b^)	0.06 (0.02, 0.18)	< 0.001
Age (years)	1.00 (0.96, 1.05)	0.854
Baseline tumor size (mm)	1.00 (0.96, 1.03)	0.786
Grade		
1 versus 3	0.44 (0.17, 1.09)	0.074
2 versus 3	0.81 (0.29, 2.19)	0.671

*CI* confidence interval

*p value based on the profile likelihood test

^a^*ESR1, PGR*, and ER gene-group were not included in the prespecified multivariable analysis because they represent individual gene components of the Recurrence Score algorithm

^b^Statistics were calculated per 50-unit change in Recurrence Score result to facilitate comparison with results of the original validation studies

**Table 3:** Multivariable analysis of clinical response, controlling for clinical factors (*N* = 281)

Model	Variable	Odds ratio (95% CI)	*p* value*
1	*ESR1* by RT-PCR (standardized)	1.29 (1.00, 1.68)	0.047
	Age (years)	1.02 (0.97, 1.06)	0.470
	Baseline tumor size (mm)	0.99 (0.96, 1.03)	0.696
	Grade		
	1 versus 3	0.76 (0.33, 1.76)	0.522
	2 versus 3	0.99 (0.38, 2.54)	0.981
2	*PGR* by RT-PCR (standardized)	1.98 (1.52, 2.63)	< 0.001
	Age (years)	1.01 (0.96, 1.05)	0.803
	Baseline tumor size (mm)	1.00 (0.96, 1.04)	0.976
	Grade		
	1 versus 3	0.77 (0.32, 1.83)	0.545
	2 versus 3	1.17 (0.44, 3.12)	0.758
3	ER gene-group score (standardized)	2.05 (1.54, 2.78)	< 0.001
	Age (years)	1.01 (0.96, 1.05)	0.755
	Baseline tumor size (mm)	1.00 (0.97, 1.04)	0.874
	Grade		
	1 versus 3	0.57 (0.24, 1.38)	0.216
	2 versus 3	0.86 (0.32, 2.30)	0.759

*CI* confidence interval, *ER* estrogen receptor, *RT-PCR* reverse transcription-polymerase chain reaction

*p value based on the profile likelihood test

### Association of Recurrence Score Group With BCS candidacy before and rate of BCS after neoadjuvant letrozole

Patients in the RS < 18 group were more likely than patients in the RS ≥ 31 group to convert from BCS noncandidates to BCS recipients following neoadjuvant letrozole. Recurrence Score group was not associated with BCS candidacy before treatment (*p* = 0.878; Table 4). After neoadjuvant letrozole treatment, however, Recurrence Score group was significantly associated with BCS received (*p* = 0.009). Among patients with RS < 18 and both pre- and post-treatment data available, pre-treatment surgery recommendation significantly differed from post-treatment surgery received (McNemar’s test *p* < 0.001), whereas there was no significant change among patients with RS ≥ 31 (*p* = 0.075; Supplemental Table 2).

**Table 4:** Association of Recurrence Score group with breast-conserving surgery candidacy before and breast-conserving surgery received after neoadjuvant letrozole

Recurrence Score group	No	Yes	*p* value*
BCS candidacy before neoadjuvant letrozole			
RS < 18	60 (38%)	97 (62%)	0.878
RS ≥ 31	20 (37%)	34 (63%)	
BCS received^a^ after neoadjuvant letrozole			
RS < 18	31 (21%)	118 (79%)	0.009
RS ≥ 31	19 (40%)	29 (60%)	

*BCS* breast-conserving surgery, *RS* Recurrence Score result

*p value based on the *χ*^2^ test

^a^Among patients with nonmissing information on surgery received

## Discussion

We report the findings of TransNEOS, the largest study to date to evaluate the relationship between Recurrence Score results and clinical response to neoadjuvant hormonal therapy. Recurrence Score group was shown to be significantly associated with clinical response to neoadjuvant letrozole, meeting the prespecified primary endpoint of the study. Among patients with large tumors (≥ 2 cm), 54% of those with RS < 18 achieved CR or PR with neoadjuvant letrozole, and 79% were BCS recipients, including many who were BCS noncandidates before neoadjuvant treatment. In contrast, patients with RS ≥ 31 had a higher rate of PD with neoadjuvant letrozole. (Patients in TransNEOS with PD on neoadjuvant letrozole were discontinued and subsequently received chemotherapy of physician’s choice, either before or after surgery.) Multivariable analyses showed that the Recurrence Score result significantly predicted clinical response to neoadjuvant hormonal therapy, even after adjustment for clinical covariates (age, tumor size, and tumor grade). Tumor grade, Ki-67 by IHC, and proliferation gene-group score were not predictive of clinical response to neoadjuvant letrozole. Our study thereby validates the Recurrence Score test as a predictor of clinical response to six months of neoadjuvant letrozole in postmenopausal patients with ER+, HER2-negative, clinically node-negative, non-metastatic primary early breast cancer.

Validation of the Recurrence Score test as a predictor of clinical response to neoadjuvant hormonal therapy represents an important milestone for patients with large, hormone receptor-positive (HR+) breast tumors. Our findings support the utility of the Recurrence Score test to identify patients with HR + breast cancer who may be BCS noncandidates initially because of large tumor size but may convert to BCS candidates with neoadjuvant hormonal therapy. This could minimize exposure of patients to chemotherapy and its associated toxicities. The TransNEOS findings complement those of previous studies suggesting that the Recurrence Score test may help guide decisions about neoadjuvant chemotherapy [27–33]. In the Pivot and Yardley studies of neoadjuvant chemotherapy, the rate of pathologic CR was 26% (*p* = 0.02) and 30% (*p* = 0.002), respectively, among patients with RS ≥ 31 but 0% among patients with RS < 18 [30, 31]. Recently, Bear and colleagues assessed the feasibility of using Recurrence Score results to guide neoadjuvant systemic therapy [27]. Patients with large (≥ 2 cm) HR+, HER2-negative breast cancers who were BCS noncandidates were assigned neoadjuvant therapy based on Recurrence Score results: RS < 11 received hormonal therapy, RS ≥ 26 received chemotherapy, and RS11–25 were randomized to hormonal therapy or chemotherapy. Rates of clinical response (CR + PR) were significantly associated with Recurrence Score group (*p* = 0.049): 83% for RS < 11 (hormonal therapy alone), 50% for RS11–25 (hormonal therapy), 73% for RS11–25 (chemotherapy), and 93% for RS ≥ 26 (chemotherapy). Rates of successful BCS after neoadjuvant systemic therapy were not significantly different across Recurrence Score groups: 75% for RS < 11, 72% for RS11–25 (hormonal therapy), 64% for RS11–25 (chemotherapy), and 57% for RS ≥ 26 [27]. Previously published data on the Recurrence Score test in neoadjuvant chemotherapy studies [27–33] coupled with our findings support the clinical validity of the Recurrence Score test to guide neoadjuvant treatment selection of hormonal therapy or chemotherapy in postmenopausal patients with locally advanced HR + breast cancer to maximize clinical response and likelihood of BCS.

The Recurrence Score test has been repeatedly validated to predict adjuvant chemotherapy benefit [21–26]. Notably, the TAILORx (Trial Assigning IndividuaLized Options for Treatment) confirmed in the largest-ever prospective, phase 3, randomized, controlled trial in breast cancer the prognostic and predictive utility of the Recurrence Score test for node-negative breast cancer in the adjuvant setting [25]. Now the test is also validated to predict response to neoadjuvant hormonal therapy, thus demonstrating consistency in the capacity of the Recurrence Score test to guide systemic treatment decisions in both the neoadjuvant and adjuvant settings. A recent meta-analysis showed that 15-year rates of distant recurrence and breast cancer-specific mortality, but not local recurrence, were comparable between patients who received chemotherapy in the neoadjuvant vs. adjuvant setting [37]. Therefore, the ability of clinicians to assess tumor biology earlier, at the time of core-biopsy sampling, may allow optimization of subsequent systemic treatment decisions. Along these lines, our study demonstrates the feasibility of performing Recurrence Score testing in core-biopsy samples and contributes to a body of evidence showing the practicality of using such specimens for testing [27, 31, 38, 39]. In TransNEOS, 38 of 333 (11.4%) samples were excluded from the analysis, including 34 (10.2%) for specimen-related reasons. This exclusion rate is similar to the 8.8% specimen-related failure rate observed in an analysis of core-biopsy samples tested in routine practice [38]. Use of core-biopsy samples for Recurrence Score testing may shorten the time to treatment decisions. In a recent study of early guideline-directed Recurrence Score testing, 94% of samples tested were core biopsies. Median time to treatment decision was shortened from 32 to 20 days (*p* < 0.001) [39]. As such, the feasibility of Recurrence Score testing on core-biopsy samples has the potential to minimize the lag between diagnosis and treatment initiation, although further studies are needed to determine the effect of this on clinical outcomes.

Our findings warrant further investigation of the Recurrence Score results in the neoadjuvant setting to address potential limitations. First, TransNEOS was a prospectively designed study of archived tumor samples. Bear and colleagues have already demonstrated the feasibility of using Recurrence Score results to assign neoadjuvant treatment in a prospective manner [27]. Second, the TransNEOS cohort was constrained geographically, which carries implications for the generalizability of the findings, with respect to racial/ethnic diversity of the patient population and country-specific clinical best practices. Third, patients were selected from the 25 highest enrolling centers only. Fourth, this study did not collect information on BCS candidacy after neoadjuvant letrozole, only actual surgery received. Some patients may have been BCS candidates after neoadjuvant treatment but received mastectomy nonetheless because of patient preference or other reason(s). Fifth, the role of the Recurrence Score test in premenopausal patients in the neoadjuvant setting was not evaluated in TransNEOS. Results of TAILORx (adjuvant setting) showed that patients ≤ 50 years and RS < 11 or RS11–15 had good outcomes with endocrine therapy alone [25]. This suggests that the genomic information provided by Recurrence Score test might have utility in guiding treatment decisions in premenopausal women who are candidates for neoadjuvant therapies. Further investigations would be needed to conclude definitively.

In conclusion, TransNEOS validates the Recurrence Score result as a significant predictor of clinical response to neoadjuvant letrozole in postmenopausal women with ER+, HER2-negative, clinically node-negative breast cancer. Additional analyses may be conducted to examine the relationship between the Recurrence Score results and clinical outcomes once the parent NEOS study results become available.

## Electronic supplementary material

Below is the link to the electronic supplementary material.

Supplementary material 1 (DOCX 52 KB)

## Data Availability

The datasets generated and/or analyzed for the current study are not publicly available, because of the proprietary information contained within, but are available from the corresponding author on reasonable request.

## Abbreviations

BCS: Breast-conserving surgery
CI: Confidence interval
CR: Complete response
ER: Estrogen receptor protein
*ESR1*: Estrogen receptor 1 gene
HER2: Human epidermal growth factor 2 protein
IHC: Immunohistochemistry
MRI: Magnetic resonance imaging
NEOS: New Primary Endocrine Therapy Origination Study
NSABP: National Surgical Adjuvant Breast and Bowel Project
PD: Progressive disease
*PGR*: Progesterone receptor gene
PgR: Progesterone receptor protein
PR: Partial response
RS: Recurrence Score result
RT-PCR: Reverse transcription-polymerase chain reaction
SD: Stable disease
st dev: Standard deviation
