# Supplementary material for: Validation of the 21-gene test as a predictor of clinical response to neoadjuvant hormonal therapy for ER+, HER2-negative breast cancer: the TransNEOS study
Source: Breast Cancer Res Treat. 2018 Sep 21;173(1):123–33. doi: 10.1007/s10549-018-4964-y (PMC6394785; doi:10.1007/s10549-018-4964-y)

**Supplemental Materials**

**Supplemental Table 1.** Univariable Analysis of Clinical Response.

| **Variable** | | **N** | **Odds Ratio (95% CI)** | **P-value*** |
| --- | --- | --- | --- | --- |
| Recurrence Score result (50 units^a^) | | 295 | 0.07 (0.02,0.21) | <0.001 |
| Age (y) | | 295 | 1.01 (0.98, 1.06) | 0.467 |
| Baseline tumor size (mm) | | 295 | 0.99 (0.95, 1.02) | 0.439 |
| Grade | | 281 |  | 0.594 |
|  | 1 vs. 3 |  | 0.83 (0.37, 1.88) |  |
|  | 2 vs. 3 |  | 1.11 (0.45, 2.79) |  |
| *ESR1* by RT-PCR | | 295 | 1.25 (1.04, 1.52) | 0.019 |
| *PGR* by RT-PCR | | 295 | 1.42 (1.25, 2.28) | <0.001 |
| ER gene-group score | | 295 | 1.80 (1.44, 2.28) | <0.001 |
| Ki-67 by IHC (%) | | 270 | 0.99 (0.97, 1.00) | 0.137 |
| Proliferation gene-group score | | 295 | 1.00 (0.70, 1.42) | 0.981 |

^a^Statistics were calculated per 50-unit change in Recurrence Score result to facilitate comparison with results of the original validation studies.

*P-value based on the profile likelihood test.

CI, confidence interval; ER, estrogen receptor; IHC, immunohistochemistry; RT-PCR, reverse transcription-polymerase chain reaction.

**Supplemental Table 2.** Change in surgery by Recurrence Score Risk Group.

|  | **Pre-treatment Surgery Candidacy** | **Post-treatment Surgery Received** | **n (%)** | **P-value*** |
| --- | --- | --- | --- | --- |
| **RS<18** | BCS | BCS | 85 (57.1%) | <0.001 |
|  |  | Mastectomy | 7 (4.7%) |  |
|  | Mastectomy | BCS | 33 (22.2%) |  |
|  |  | Mastectomy | 24 (16.1%) |  |
| **RS≥31** | BCS | BCS | 22 (45.8%) | 0.075 |
|  |  | Mastectomy | 6 (12.5%) |  |
|  | Mastectomy | BCS | 7 (14.6%) |  |
|  |  | Mastectomy | 13 (27.1%) |  |

*P-value by McNemar’s test.

**Supplemental Figure 1.** NEOS Parent Study Schema. *Stratification by response to neoadjuvant letrozole (CR or PR vs. SD); PgR status (positive vs. negative); nodal status (positive vs. negative); age (<60 vs. ≥60 y); study center. CR, complete response; ER, estrogen receptor; HER2, human epidermal growth factor 2; PD, progressive disease; PgR, progesterone receptor; PR, partial response; SD, stable disease.

**Supplemental Figure 1**
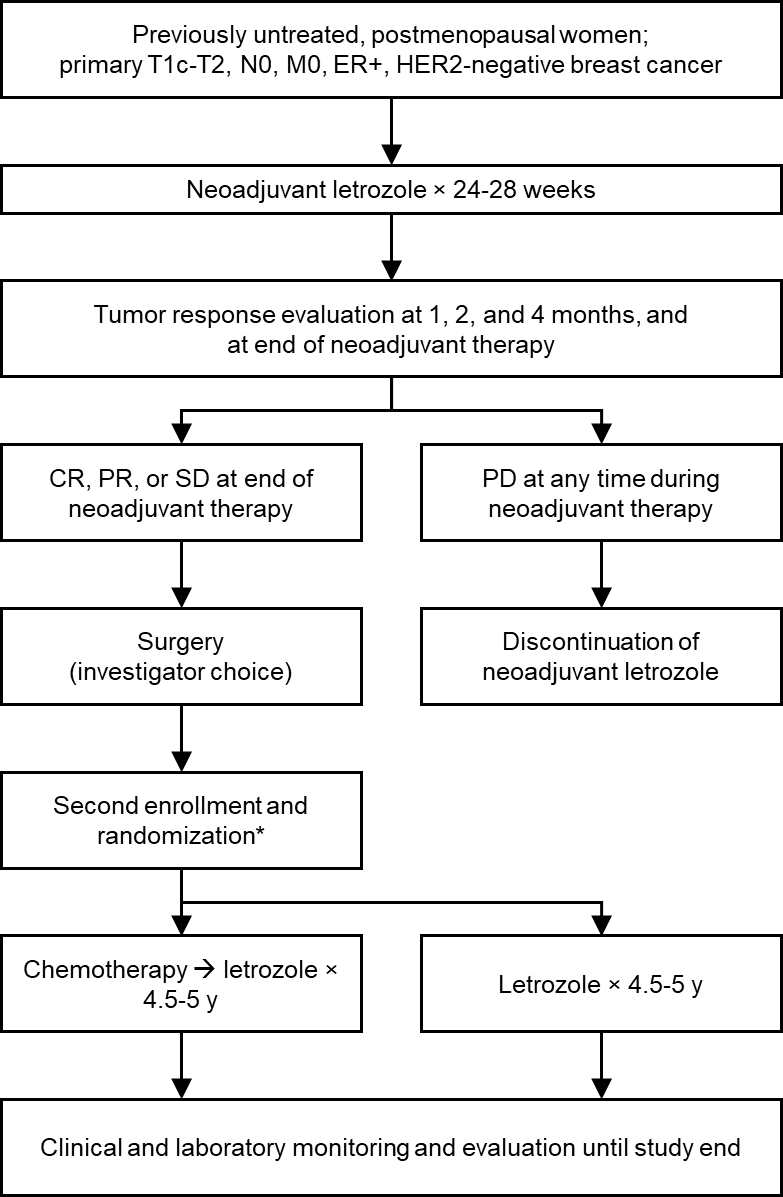

Supplement: Supplementary file 1 — Supplementary material 1 (DOCX 52 KB) [file 10549_2018_4964_MOESM1_ESM.docx]
